# Predatory publishing in medical education: a rapid scoping review

**DOI:** 10.1186/s12909-024-05024-x

**Published:** 2024-01-05

**Authors:** Owen W Tomlinson

**Affiliations:** Department of Clinical and Biomedical Science, Faculty of Health and Life Science, University of Exeter Medical School, University of Exeter, Exeter, UK

**Keywords:** Publishing, Review, Students, Information literacy, Undergraduate

## Abstract

**Background:**

Academic publishing is a cornerstone of scholarly communications, yet is unfortunately open to abuse, having given rise to ‘predatory publishers’– groups that employ aggressive marketing tactics, are deficient in methods and ethics, and bypass peer review. Preventing these predatory publishers from infiltrating scholarly activity is of high importance, and students must be trained in this area to increase awareness and reduce use. The scope of this issue in the context of medical students remains unknown, and therefore this sought to examine the breadth of the current literature base.

**Methods:**

A rapid scoping review was undertaken, adhering to adapted PRISMA guidelines. Six databases (ASSIA, EBSCO, Ovid, PubMed, Scopus, Web of Science) were systematically searched for content related to predatory publishing and medical students. Results were single-screened, facilitated by online reviewing software. Resultant data were narratively described, with common themes identified.

**Results:**

After searching and screening, five studies were included, representing a total of 1338 students. Two predominant themes– understanding, and utilisation– of predatory publishers was identified. These themes revealed that medical students were broadly unaware of the issue of predatory publishing, and that a small number have already, or would consider, using their services.

**Conclusion:**

There remains a lack of understanding of the threat that predatory publishers pose amongst medical students. Future research and education in this domain will be required to focus on informing medical students on the issue, and the implication of engaging with predatory publishers.

**Supplementary Information:**

The online version contains supplementary material available at 10.1186/s12909-024-05024-x.

## Introduction

Academic publishing is a fundamental cornerstone of scholarly communication, whereby research discoveries are shared with the global academic community via peer review– a process that independently validates findings and ensures research quality [1]. Publishing in peer-reviewed journals (those corroborated by academic peers) can also establish and consolidate credibility of individual researchers, and therefore individuals (e.g., researchers, lecturers, practitioners, students) are often encouraged to publish widely as part of their careers, and do so for multiple reasons [2].

However, this valuable sector is subsequently liable to risk and fraud, with the rise of illegitimate publishers (also referred to as ‘potentially predatory publishers’ [3]); groups and companies that proclaim to be academic publishers, yet deviate from best editorial practice. These illegitimate publishers often lack transparency, contain false or misleading information, employ aggressive solicitation tactics to obtain submissions [4], and even engage in elements of cyber-criminality [5]. These illegitimate publishers often charge authors large open access fees for publishing rights, often accompanied by a promise of a quick production timeline [6].

Whilst the operating practices of these illegitimate publishers vary, there are several characteristics by which they can be identified. These include deceptive practices, fake impact factors, no retraction policies, unclear contact details, non-verifiable affiliations for editors, and low transparency surrounding its publishing operations [7]. Thus, whilst a universal definition is lacking, the term ‘predatory publisher’ has become synonymous with the practice and is therefore used herein [4].

Articles published by predatory publishers will often be deficient in reporting of methods and lack ethical approval to take place [8], exhibit plagiarism [9], avoid rigorous peer review [10] and thus fundamentally erode the credibility of the literature base [11]. It has also been proposed that willingly submitting to such publishers may be construed as a form of academic misconduct [10], and therefore should be avoided by academics at all levels. The prevalence of predatory publishers and their outputs has increased over recent years [12], with some even beginning to infiltrate biomedical databases [13], and attract citations in student bibliographies [14] as well as the wider literature base [15].

This infiltration into databases and the wider literature has a notable potential to impact fields of study (and practice) allied to health and medicine, whereby apparent findings may be accidentally translated into patient care, having a negative impact upon all involved [11]. Therefore, additional measures are likely needed within medicine and associated fields to ensure trainees and practitioners are aware of such predatory publishers, thus offsetting the risk they bring to medical research [3]. There is an apparent awareness of predatory publishers amongst some medical faculty [16] and senior academics [17] which will help to offset this risk, yet there remains little information on whether medical students– trainees starting their clinical and research careers– are aware of the concept of predatory publishers.

Medical students are a group that are likely to participate in research as part of their studies and training [18], whereby intrinsic interest in performing research as part of their studies is present [19]. However, extrinsic motivators will be predominantly responsible, as surveys indicate that ‘increasing employability’ [20] and ‘career progression’ [21] are driving factors for engaging in research amongst medical students of all years. This in turn is driven by the high value placed upon research and publications for selection into residency programmes in the United States [22] and specialty training pathways in the United Kingdom [23]. Students who undertake research as undergraduates are more likely to be research active in their careers [24], publish their work [25, 26] and achieve both short-term and long-term successes [27].

To achieve such success, students will require journals to publish such work, whereby prior surveys have noted that students have submitted articles to journals that will have a “high likelihood of manuscript acceptance” [21]. Thus, in their desire to be published for the numerous aforementioned reasons, this cohort of students will be particularly susceptible to the risk of predatory publishers and therefore understanding the current literature base surrounding predatory publishing surrounding medical students is of utmost importance.

Therefore, the purpose of this review was to identify the breadth and depth of the current literature base in relation to predatory publishing and medical students. Results and findings will be able to provide insight and direction into the current status of the field, and identify future research and education directions to ensure medical students are equipped to mitigate this scholarly phenomenon.

## Methods

### Review strategy

This scoping review is undertaken and reported in accordance with the reporting guidance provided in the ‘PRISMA-ScR’ (Preferred Reporting Items for Systematic reviews and Meta-Analyses extension for Scoping Reviews) checklist [28].

A scoping review was opted for as opposed to a systematic review due to unknown nature of the existing literature in this domain, and to therefore examine the extent and range of research activity in this domain, and summarise primary findings, whilst determining if a full systematic review and meta-analyses will be warranted [29]. Moreover, a ‘rapid’ review was performed due to resource limitations (i.e., single reviewer and author), aligning with existing definition for a rapid review [30].

### Eligibility criteria

Studies were limited to those published in English, with no restrictions being placed on publication dates. Studies were included in this scoping review based upon a series of pre-planned inclusion criteria.

This review focuses on undergraduate medical students only. Individuals who had already graduated were excluded, even if they were undertaking further medical training. Individuals training for similar degrees (e.g., nursing, dentistry, medical science) were excluded, as were individuals where ambiguity existed on their status (i.e., general mentions of ‘students’ were not included). There was no limitation upon nationality of medical students themselves, or location of medical training.

In addition, this scoping review sought to only include original research (i.e., excluding reviews, letters, opinions), but did not discriminate on research design or quality. All research related to predatory publishing was included, provided it was in relation to the aforementioned population (medical students). If studies included medical students as part of a wider sample, these were only carried forward for inclusion on the provision that findings and data from this sub-group could be explicitly identified (i.e., pooled analyses and studies were excluded).

### Information sources

Six databases were searched, each from their own inception, up until August 2023: (1) PubMed, (2) Scopus, (3) Web of Science, (4) Ovid (Ovid MEDLINE, APA PsychInfo, Embase, Social Policy and Practice, Global Health, CAB Abstracts, HMIC Health Management Consortium, APA PsychExtra), (5) EBSCO (CINHAL Ultimate, AMED, ERIC, MEDLINE), and (6) Applied Social Sciences Index & Abstracts.

In addition, forward and backward citation searches of included studies was undertaken by hand to identify additional sources that may have been omitted by original searches. No searches of the grey literature were made.

### Search terms

The following terms (and associated Boolean operators) were used to undertake searches: (predatory journal OR predatory publish*) AND (medical OR medicine) AND (student). This was designed to be as broad as possible, encapsulating as many returns as possible for entries related to medical students, specifically. A full breakdown of all search strategies is provided in **Supplemental File 1**.

### Selection of sources

Records were imported and managed via online evidence synthesis software (Covidence systematic review software, Veritas Health Innovation, Melbourne, Australia). A single researcher screened all articles, in a two-step process. First, titles and abstracts of identified papers were assessed in relation to aforementioned eligibility criteria. Secondly, eligible articles had full-texts retrieved and then screened in full, again against the aforementioned eligibility criteria.

### Data extraction, appraisal & synthesis

The following data items were extracted from each study: (1) Study Design & Purpose; (2) Study Population & Setting; (3) Summary of Key Findings. A single author extracted these, with information displayed in Table 1. Included studies were narratively synthesised, with key findings of each study discussed via thematic approaches. No formal risk of bias or statistical analyses were undertaken.

**Table 1:** Included studies in accordance with inclusion and exclusion criteria

Study	Study Design & Purpose	Population & Setting	Key Finding(s)
Abu-Zaid, 2019 [35]	Development of ‘advisory peer review board’ by medical graduate to aid in dissemination of medical student research in ‘mainstream’ journals.	82 ‘student authors’. Setting and nationality not specified ^a^.	Intervention *“reduced the likelihood”* of students publishing in predatory journals.
Alamri et al., 2020 [31]	Survey of students to identify (amongst other outcomes) awareness of predatory journals.	198 medical students from Saudi Arabia (61.6% female). 65 medical students from New Zealand (64.6% female).	Minority of students from both countries familiar with the term ‘predatory journal’ (9.1% Saudi Arabia vs. 7.8% New Zealand). 7/31 publications by students were in predatory journals.
Ashour & Funjan, 2022 [32]	Survey of students information literacy, including impressions and attitudes towards predatory publishers.	195 medical students from Jordan (56.9% female).	20% of students would read contents of a journal article without verifying its reliability (i.e., predatory or non-predatory).
Kabulo et al., 2022 [33]	Survey of knowledge, exposure to, and intention to submit to predatory journals.	101 neurosurgeons from multiple countries in Africa. 28/101 (27.7%) students.	No impact of professional level (consultant/resident/student) upon rate of publishing in predatory journals. 2/28 (7%) of students would submit to predatory journals ^b^.
Nicolalde et al., 2022 [34]	Survey of scientific literacy, including ability to identify concept of a predatory publisher.	770 medical students from Latin America (63.6% female).	243 (31.6%) correctly identified characteristics of predatory journals.

Location is presumed to be United States of America as the corresponding author gives this address within the publication. b) Possible reporting error in article, as number of students in authors Table 1 (*n* = 28) contradicts the number in authors Table 2 (*n* = 34) [33]

## Results

**Fig. 1:**
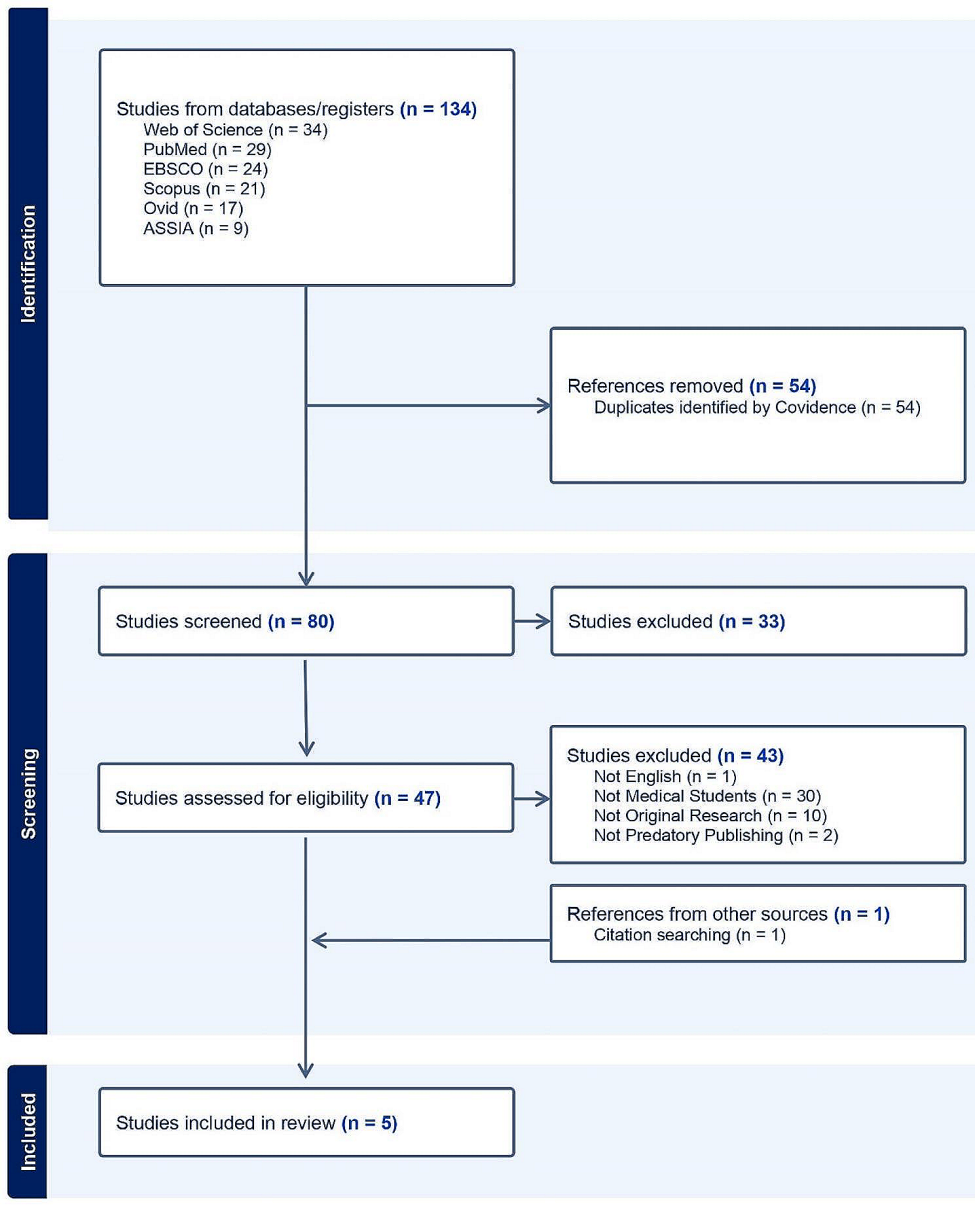
PRISMA flow chart detailing inclusion of studies for final scoping review. Reasons for exclusion are not mutually exclusive and therefore articles may be excluded for more than one reason. ASSIA: Applied Social Sciences Index & Abstracts; PRISMA: Preferred Reporting Items for Systematic reviews and Meta-Analyses

A total of 134 studies were identified from searches, 80 of which were title-screened. Of these, 47 studies underwent full-text screening. Of these, 43 were excluded for various reasons (**Supplemental File 2**) and thus four were included in the final sample, alongside a single study identified via hand-searches (Fig. 1).

The resultant studies and their characteristics are listed in Table 1. A total of 1338 students, from five continents are represented; none were from Europe. Four of the included studies were surveys, focusing on awareness and understanding of predatory publishers [31–34], with one assessing an intervention [35]. When considering the findings of each study, these can be grouped into two overarching themes: *understanding*, and *utilisation*.

### Understanding

Three of the survey studies [31, 33, 34] asked participants about their awareness and understanding of predatory publishers.

In the first of these from Alamri et al., [31], a minority of medical students (7.8– 9.1%) were familiar with the term ‘predatory journal’, whilst the majority were ‘unsure’ about their characteristics. This included whether journals who (a) lacked an impact factor, (b) were not indexed in PubMed, (c) charge article processing fees, and (d) based in developed countries, should– or should not– be defined as ‘predatory’, with > 70% of all respondents being ‘unsure’ in these situations. Finally, only 5/263 students in the study believed it was “easy” to differentiate predatory from legitimate open-access journals.

In a second study from Nicolalde et al., [34], 31.6% of students could correctly identify the characteristic of a predatory journal. Moreover, a significant difference was noted between in the prevalence of those who attended public vs. private universities (37.1% vs. 26.0%), but not stage of medical school (years 1 and 2 vs. years 3 and 4), in their ability to identify the concept of a predatory publisher.

A third survey from Kabulo et al., [33], asked questions of its respondents regarding their confidence in identifying a predatory publisher, but did not provide any sub-group data specifically pertaining to medical students.

### Utilisation

Three of the survey studies [31–33] included data regarding: (a) use of journals from predatory publishers, and the information within the articles, and (b) use of predatory publishers (via submission and publication) to convey findings. A further study identified a method to prevent use of predatory publishers to disseminate results [35].

Firstly, in the survey of Ashour & Funjan [32], it was noted that 39/195 (20.0%) of students would read literature found online without confirming the reliability of the journal (i.e. predatory vs. non-predatory). This is in contrast to the majority of students (116/195; 59.5%) who would only read an article after ensuring it is from a reliable source.

Secondly, the survey from Kabulo et al., [33] identified that a small proportion of students (2/28; 7.1%) intended to submit to a predatory publisher in the future– a similarly small proportion to consultants (1/39; 2.6%) and residents (4/34; 11.8%).

Thirdly, Alamri et al., [31] included the finding that of the students who had already published as part of their studies, a small proportion had done so in predatory journals. This included 6/23 (26.1%) of students in Saudi Arabia, and 1/8 (12.5%) student in New Zealand; a non-statistically significant difference between nations.

Finally, a brief interventional approach described by Abu-Zaid [35] indicated that having an advisory peer-review board– handling research, provision of critical evaluation and suggestions, and guiding formatting– prior to submission to actual journals *“reduced the likelihood of publishing in MSJs [medical student journal] or predatory journals”*, although no formal statistics were provided on this likelihood.

## Discussion

The aim of this scoping review was to ascertain the current status of the evidence base in relation to medical students and predatory publishing. The results of this review have indicated that two predominant themes are present within the literature– *understanding* of predatory publishing, and *utilisation* of their outputs.

Interestingly, only five studies were eligible for inclusion within this scoping review. Whilst many results were obtained via the search strategy, many were excluded as they either did not refer to medical students, or were not original research, amongst other reasons. Several studies in this area of research area refer to ‘students’ without clarifying whether they are in fact medical students [36], some report on specific medical specialties [37–39], students in aligning fields [40], graduates [41], and allied health professions [42, 43]; yet few focused on those in their formative years of becoming registered medical doctors.

Whilst this paucity of research could initially be considered a concerning finding, it should be noted that the phenomenon of predatory publishing is relatively recent. First descriptions of predatory publishing emerged ~ 15 years ago [44], with editorials [45] and quasi-experimental descriptions [46] occurring ~ 5 years thereafter. Therefore, this relatively recent occurrence may explain the relatively low level of reporting and research in relation to medical students.

### Understanding of predatory publishing

The first of the two predominant themes identified was that of *understanding* predatory publishers. Cumulatively, the included studies identified that relatively few students were aware of the concept of predatory publishers, nor were able to identify characteristics of predatory publishers [31, 33, 34]. The highest awareness came from Nicolalde et al., whereby ~ 30% of students could identify characteristics of a predatory journal [34].

In contrast to this relatively low rate of awareness amongst students, the understanding of predatory publishers has been reported to be higher amongst groups elsewhere in medicine. For example, medical faculty have a relatively high awareness (> 70%) and ability to identify such publishers [47], although this rate then decreases in oncology specialists (47.8%) [38] and dermatology specialists (20.3%) [39]. Moreover, identifying the individual characteristics of predatory publishers is variable amongst medical residents [48] and therefore the ambiguity in understanding and identifying predatory publishers may not be confined to medical students.

The issue of how best to define (and thus identify, and understand) a predatory publisher has been a matter of debate, as no uniform definition exists. Initial criteria [49] and lists of predatory publishers [50] have been of assistance, but these are still unknown by many clinicians [48]. Moreover, notable variance has been reported between empirical studies examining this phenomenon [51], leading to multiple checklists being developed [52], although these are purportedly of questionable validity and reliability [53]. In attempting to resolve some of this ambiguity, an expert consensus document has been developed, with characteristics, markers and empirically derived data that can be used to differentiate predatory from legitimate publishers [7].

In addition to checklists, separate ‘whitelists’ and ‘blacklists’– lists of ‘validated’ or ‘illegitimate’ journals– have also been developed to aid in this decision-making process [54]. However, analyses indicate there are some journals that exist on both sets of lists [55], and thus their true status as predatory or not, is unknown. This in turn creates the concept of an ambiguous ‘grey zone’ in publishing, whereby academics, clinicians and mentors must be aware, yet whereby academic librarians are also primely positioned to aid in this education regarding predatory publishing [56, 57].

### Utilisation of predatory publishers

The second of the themes identified in this review was that of *utilisation* of predatory publishers. This included students in the study of Ashour & Funjan [32] who stated they would read contents of journals (including predatory publications) without verifying the reliability of the source, thus using the outputs of predatory journals. In addition, students within the work of Kabulo et al., stated they would submit to such predators for publication [33], and students in Alamri et al., who stated that had already done so [31].

Therefore, within this theme, it can be observed that ‘utilisation’ takes multiple forms, all of which are cause for concern. In using the outputs from predatory publishers, this lends validity to the predators, enhancing their air of legitimacy. Outputs from predatory publishers are beginning to infiltrate bibliographic databases [13, 58], attract citations from student bibliographies [14] and the wider academic community [15, 59], with some analyses indicating that > 40% of predatory articles have been attracted more than one citation [15].

Whether articles published by predatory publishers should be included in syntheses of evidence is a topic that is widely discussed [60–62]. Specialists in evidence synthesis techniques suggest that such articles from may in fact be suitable for inclusion in synthesis projects on the provision that studies are of ‘high quality’ and results are independently verified [60], with additional reporting guidelines [61] and sensitivity analyses [61, 62] also being proposed as potential mechanisms to maintain academic standards.

In addition to using predatory articles to inform research practice, some authors also explicitly and wilfully submit work to these predators for publication. As to why this happens, many reasons may be present [63]. An element of naïvety will be responsible– as shown by students in this review who were unaware of the concept of predatory publishers [31, 34]. In addition, factors such as pressure to publish from host institutions, lack of academic proficiency, promises of rapid publication, social identity threats, and the tactics employed by predatory publishers [17, 64–67] can all result in continued submissions.

### Educating students

Given the aforementioned understanding (or lack thereof) and utilisation of predatory publishers by medical students it is evident that training and education on the topic will become necessary for this group to prevent adding further legitimacy to these publishers and erosion of the literature base. As noted previously, use of checklists [52], and both whitelists and blacklists [54], may help in the decision making process for students, when understanding what may constitute a predatory publisher. However, students must first be aware of predatory publishers before these lists become functionally useful. They must be taught, guided and mentored by senior academics and clinicians to ensure they understand what these predators are, how they operate, and how to avoid them.

Therefore, specific interventions aimed at developing understanding of the publishing process, including reference to predatory publishers should be developed. Previous ‘information workshops’ containing content on predatory publishers have been designed and effectively implemented for medical residents and graduate trainees [68, 69], nursing students [70], allied health professionals [71] and veterinary trainees [16]. These workshops increase awareness and improve knowledge in this important field of scholarly activity.

Whilst such workshops or information interventions have yet to be developed explicitly for medical students (as none were found for inclusion in the current review), the principles and underlying information in existing interventions may feasibly be adapted for future use. Therefore, future studies should urgently design novel workshops (or adapt/replicate existing ones), for implementation and evaluation in this group of students.

### Strengths & limitations

There are several strengths, and some limitations, to report within this scoping review. Whilst the issue of predatory publishing has been studied in the context of medical students previously (as shown by the studies identified for inclusion within the present review), this current study presents the first time this concept has been examined holistically within this group, pooling all available original research on the topic. In addition, the systematic searching strategy, utilising multiple databases and existing frameworks [28], serves to enhance the rigour of the methodology and enhancing confidence in the final results.

In contrast, it could be argued that use of an explicit framework such as that of Arksey & O’Malley [29] could prove more robust, although in retrospect this review does align with the principles laid down by this framework and closely follows explicit steps (although not all) provided. Moreover, use of a single reviewer may within the screening process may result in some missed articles, although the rate of omission can be low with experienced researchers [72]. However, given the small number of studies to be identified and screened, it is unclear whether a double-screening process would have affected the final results and interpretation, particularly considering the relatively high proportion of excluded studies that were not original research. This use of a single screener directly links to the decision to badge this study as a rapid review, whereby a streamlining of resources (alongside acceleration of processes) is the fundamental condition of making a rapid review [30]. Whilst exact methods for conducting a rapid review are variable, many are in agreement that use of a single researcher for screening and/or extraction tasks would constitute a rapid review [73].

## Conclusion

In summary, this scoping review has for the first time, holistically examined the prevalence and depth of research in relation to predatory publishers within the context of medical students. This has identified two predominant themes, those of *understanding* the issue of predatory publishing, and *utilisation* of their outputs and services. This review had identified a broad lack of awareness and understanding about the issue, yet also a small proportion of medical students that would willingly engage with predatory publishers, despite the risk they pose. Future research and education in this area will need to focus on informing students about the threat predatory publishers present to academia, and clinical decision making. Whilst there will remain an onus on individual students, researchers, and clinicians to avoid predatory publishers, there is also scope for a wide body of stakeholders– including funding agencies and the wider higher education sector– to aid in the resolution of this issue [74].

### Electronic supplementary material

Below is the link to the electronic supplementary material.

**Supplementary Material 1**: Search strategies utilised within each database

**Supplementary Material 2**: Excluded studies

## Data Availability

All data pertaining to this study is presented within the manuscript.
