# Supplementary material for: Predatory publishing in medical education: a rapid scoping review
Source: BMC Med Educ. 2024 Jan 5;24:33. doi: 10.1186/s12909-024-05024-x (PMC10770935; doi:10.1186/s12909-024-05024-x)
Supplement: Supplementary file 1 — Supplementary Material 1: Search strategies utilised within each database [file 12909_2024_5024_MOESM1_ESM.pdf]

Supplemental File 1. Search strategies utilised within each database.

**Database**	**Search Terms (Input by Author)**	**Number of Results**	**Query generated by Database**
PubMed	(predatory journal OR predatory publish*) AND ((medical OR medicine)) AND (student))	29	("predatory journals as topic"[MeSH Terms] OR ("predatory"[All Fields] AND "journals"[All Fields] AND "topic"[All Fields]) OR "predatory journals as topic"[All Fields] OR ("predatory"[All Fields] AND "journal"[All Fields]) OR "predatory journal"[All Fields] OR (("predatorial"[All Fields] OR "predatory"[All Fields]) AND "publish*"[All Fields])) AND ("medic"[All Fields] OR "medical"[All Fields] OR "medicalization"[MeSH Terms] OR "medicalization"[All Fields] OR "medicalizations"[All Fields] OR "medicalize"[All Fields] OR "medicalized"[All Fields] OR "medicalizes"[All Fields] OR "medicalizing"[All Fields] OR "medically"[All Fields] OR "medicals"[All Fields] OR "medicated"[All Fields] OR "medication s"[All Fields] OR "medics"[All Fields] OR "pharmaceutical preparations"[Supplementary Concept] OR "pharmaceutical preparations"[All Fields] OR "medication"[All Fields] OR "pharmaceutical preparations"[MeSH Terms] OR ("pharmaceutical"[All Fields] AND "preparations"[All Fields]) OR "medications"[All Fields] OR ("medicin"[All Fields] OR "medicinal"[All Fields] OR "medicinally"[All Fields] OR "medicinals"[All Fields] OR "medicine"[MeSH Terms] OR "medicine"[All Fields] OR "medicine s"[All Fields] OR "medicines"[All Fields])) AND ("student s"[All Fields] OR "students"[MeSH Terms] OR "students"[All Fields] OR "student"[All Fields] OR "students s"[All Fields])
Ovid (Ovid MEDLINE, APA PsycInfo, Embase, Social Policy and Practice, Global Health, CAB Abstracts, HMIC Health Management Information Consortium, APA PsycExtra)	(predatory journal OR predatory publish*) AND ((medical OR medicine)) AND (student))	17	((predatory journal or predatory publish*) and (medical or medicine) and student).af.

EBSCO (CINAHL Ultimate, AMED, ERIC, MEDLINE)	(predatory journal OR predatory publish*) AND ((medical OR medicine)) AND (student))	24<br><br>NB. exact duplicates removed from results	(predatory journal OR predatory publish*) AND (medical OR medicine) AND student
Scopus	(predatory journal OR predatory publish*) AND ((medical OR medicine)) AND (student))	21	(TITLE-ABS-KEY (predatory AND journal OR predatory AND publish*) AND TITLE-ABS-KEY (medical OR medicine) AND TITLE-ABS-KEY (student))
Web of Science	(predatory journal OR predatory publish*) AND ((medical OR medicine)) AND (student))	34	(predatory journal OR predatory publish*) (All Fields) and (medical OR medicine) (All Fields) and student (All Fields)
Applied Social Sciences Index & Abstracts (ASSIA)	("predatory journal" OR "predatory publish*") AND (medicine OR medical) AND student	9	("predatory journal" OR "predatory publish*") AND (medicine OR medical) AND student

Searches conducted on 18/08/2023.
