# Supplementary material for: Predatory publishing in medical education: a rapid scoping review
Source: BMC Med Educ. 2024 Jan 5;24:33. doi: 10.1186/s12909-024-05024-x (PMC10770935; doi:10.1186/s12909-024-05024-x)
Supplement: Supplementary file 2 — Supplementary Material 2: Excluded studies [file 12909_2024_5024_MOESM2_ESM.pdf]

Supplemental File 2 – Excluded Studies

Study	Title	Authors	Published Year	Journal	Volume	Issue	Pages	Notes
Broome 2021	Publishing in Predatory Journals: Guidelines for Nursing Faculty in Promotion and Tenure Policies	Broome, Marion E; Oermann, Marilyn H; Nicoll, Leslie H; Waldrop, Julee B; Carter-Templeton, Heather; Chinn, Peggy L.	2021	Journal of Nursing Scholarship	53	6	746-752	Exclusion reason: Not Med Students;
Oermann 2016	Study of Predatory Open Access Nursing Journals	Oermann, Marilyn H; Conklin, Jamie L; Nicoll, Leslie H; Chinn, Peggy L; Ashton, Kathleen S; Edie, Alison H; Amarasekara, Sathya; Budinger, Susan C.	2016	Journal of Nursing Scholarship	48	6	624-632	Exclusion reason: Not Med Students;
Gardenier 2015	Should Doctor of Nursing Practice Students be Required to Submit for Publication? The Faculty Perspective	Gardenier, Donald	2015	The Journal for Nurse Practitioners	11	3	298-299	Exclusion reason: Not Med Students;
Houghton 2022	Keep calm and carry on: moral panic, predatory publishers, peer review, and the emperor's new clothes.	Houghton, Frank	2022	Journal of the Medical Library Association	110	2	233-239	Exclusion reason: Not Med Students;
Al-Busaidi 2018	The hidden agenda of predatory journals: A warning call for junior researchers and student authors.	Al-Busaidi, Ibrahim Saleh; Alamri, Yassar; Abu-Zaid, Ahmed	2018	Medical Teacher	40	12	1306-1307	Exclusion reason: Not Original Research;
Wilson 2019	Predatory journals: A warning for us all...Al-Busaidi IS, Alamri Y, Abu-Zaid A. The hidden agenda of predatory journals: a warning call for junior researchers and student authors.	Wilson, David J.	2019	Medical Teacher	41	12	1452-1452	Exclusion reason: Not Original Research;
Fallik 2019	Neurology Editors Identify the Red Flags of Predatory Publishing...Michael R Sperling	Fallik, Dawn	2019	Neurology Today	19	19	58-62	Exclusion reason: Not Med Students;
Zakout 2020	Predatory Publishers/Journals in Medical Sciences: How to Avoid, Stop, and What to Do after Being Scammed by Them?	Zakout, Yosef Mohammed-Azzam	2020	Journal of Gastrointestinal Cancer	51	3	782-787	Exclusion reason: Not Med Students;
Al-Moghrabi 2023	An analysis of dental articles in predatory journals and associated online engagement.	Al-Moghrabi, Dalya; Albishri, Rana S; Alshehri, Rahaf D; Arqub, Sarah Abu; Alkadhimi, Aslam; Fleming, Padhraig S	2023	Journal of Dentistry	129		104385	Exclusion reason: Not Med Students;
Wiens 2022	Factors Affecting the Use of Medical Articles for Citation and Academic Reference.	Wiens, Ian; Ramjiawan, Angela; Wiens, Julia; Fung, Kevin; Gooi, Malcolm; Gooi, Patrick; Hu, Amanda; Leitao, Darren; Nguyen, Lily H P; Gooi, Adrian	2022	Advances in Medical Education and Practice	13		755-763	Exclusion reason: Not Predatory Publishing;

Joubert 2020	Journal response types and times: the outcomes of manuscripts finalised for submission by the University of the Free State School of Medicine medical editor, South Africa.	Joubert, Gina; Mulder, Theanette; Steinberg, Wilhelm Johannes; Botes, Johan	2020	The Pan African Medical Journal	36		212	Exclusion reason: Not Predatory Publishing;
Smith 2015	Predatory publishing houses: Challenging the legitimacy of open access journals.	Smith, Gavin	2015	Australasian Journal of Paramedicine	12	2	01-Feb	Exclusion reason: Not Med Students;
Christopher 2015	Awareness of "Predatory" Open-Access Journals among Prospective Veterinary and Medical Authors Attending Scientific Writing Workshops.	Christopher, Mary M; Young, Karen M	2015	Frontiers in Veterinary Science	2		22	Exclusion reason: Not Med Students;
Raj 2019	Plagiarism, P-hacking, and Predatory Journals: Toxic Triple Ps of Scientific Publications.	Raj, A Thirumal; Panta, Prashanth; Patil, Shankargouda	2019	The Journal of Contemporary Dental Practice	20	2	129-130	Exclusion reason: Not Original Research;
Alamri 2019	Equipping Junior Authors Against Predatory Open-Access Journals.	Alamri, Yassar; Abu-Zaid, Ahmed	2019	Academic Medicine	94	10	1405	Exclusion reason: Not Original Research;
Reves 2018	Predatory Publishing: An Industry that Is Threatening Science.	Reves, Joana; Silva, Bernardo Marques; Durao, Jose; Ribeiro, Nuno Vouga; Lemos, Samuel; Escada, Pedro	2018	Acta Medica Portuguesa	31	3	141-143	Exclusion reason: Not Med Students;
Das 2017	Publish and flourish: Take the road less travelled!	Das, A K	2017	Medical journal, Armed Forces India	73	2	178-180	Exclusion reason: Not Med Students;
Misra 2019	Open Access Publishing in India: Coverage, Relevance, and Future Perspectives.	Misra, Durga Prasanna; Agarwal, Vikas	2019	Journal of Korean Medical Science	34	27	e180	Exclusion reason: Not Med Students;
Sharma 2020	Predatory conferences in biomedical streams: An invitation for academic upliftment or predator's looking for prey.	Sharma, Hunny; Verma, Swati	2020	Saudi Journal of Anaesthesia	14	2	212-216	Exclusion reason: Not Original Research;
Klyce 2017	Junk science for sale Sham journals proliferating online.	Klyce, Walter; Feller, Edward	2017	Rhode Island Medical Journal	100	7	27-29	Exclusion reason: Not Original Research;
Shah 2022	De-naturalizing the 'Predatory': A Study of 'bogus' publications at public sector universities in Pakistan	Shah W.A.; Ali R.; Lashari A.	2022	Accountability in Research				Exclusion reason: Not Med Students;
Talari 2022	Understanding the awareness of publication ethics among medical postgraduate trainees in India: A web-based survey	Talari K.; Goyal M.	2022	Indian Journal of Rheumatology	17	6	S357-S362	Exclusion reason: Not Med Students;
Mertz 2023	Letter to the Editor: Predatory journals: Advice for plastic surgery trainees	Mertz T.; Baldwin A.J.	2023	Journal of Plastic, Reconstructive and Aesthetic Surgery	76		54-55	Exclusion reason: Not Med Students;

Akhaddar 2022	Letter to the Editor Regarding "Vulnerability of African Neurosurgery to Predatory Journals: An E-Survey of Aspiring Neurosurgeons, Residents, and Consultants"	Akhaddar A.	2022	World Neurosurgery	161		211-212	Exclusion reason: Not Original Research;
Pawar 2020	An insight into predatory journals	Pawar V.J.; Jawade J.	2020	Indian Journal of Public Health	64	1	86-89	Exclusion reason: Not Med Students;
Alahmad 2020	Critical appraisal of predatory journals in pathology	Alahmad Y.M.; Abdelhafez I.; Cyprian F.S.; Akhtar S.; Skenderi F.; Vranic S.	2020	Journal of Clinical Pathology	73	1	58-60	Exclusion reason: Not Med Students;
Gama Marques 2018	Letter to the editor: Still regarding predatory publishing	Gama Marques J.	2018	Acta Medica Portuguesa	31	4	230-231	Exclusion reason: Not Med Students;
Schwartz 2022	The savvy academic: Publishing in the social and health sciences.	Schwartz, Seth J	2022	The Savvy Academic: Publishing in the Social and Health Sciences.				Exclusion reason: Not Original Research;
West 2021	Misinformation in and about science.	West, Jevin D; Bergstrom, Carl T	2021	PNAS Proceedings of the National Academy of Sciences of the United States of America	118	5		Exclusion reason: Not Med Students;
Ortiz-Prado 2019	Predatory journals: What they are and how to avoid them	Ortiz-Prado, E.; Lister, A.	2019	Revista Ecuatoriana de Neurologia	28	1	07-Sep	Exclusion reason: Not Med Students;
Maddy 2017	Predatory journals in dermatology	Maddy, A.J.; Tosti, A.	2017	British Journal of Dermatology	177	1	307-309	Exclusion reason: Not Med Students;
Lang 2019	An approach to conference selection and evaluation: advice to avoid predatory conferences	Lang, R.; Mintz, M.; Krentz, H.B.; Gill, M.J.	2019	Scientometrics	118	2	687-698	Exclusion reason: Not Med Students;
Pawar 2018	Predatory conferences: Caveat emptor!	Pawar, M.	2018	Journal of the European Academy of Dermatology and Venereology	32	8	e308-e309	Exclusion reason: Not Med Students;
Wang 2021	Chinese PhD students' perceptions of predatory journals	Wang, J.; Xu, J.; Chen, D.	2021	Journal of Scholarly Publishing	52	2	88-106	Exclusion reason: Not Med Students;
Goyal 2020	Usefulness of a workshop on scientific writing and publication in improving the baseline knowledge deficit among postgraduates education	Goyal, M.; Dua, A.; Kedia, A.K.; Misra, D.P.; Santhanam, S.; Ravindran, V.	2020	Journal of the Royal College of Physicians of Edinburgh	50	3	316-321	Exclusion reason: Not Med Students;
Wang 2021	Chinese PhD Students' Perceptions of Predatory Journals: A Survey Study	Wang, JY; Xu, J; Chen, DY	2021	Journal of Scholarly Publishing	52	2	88-106	Exclusion reason: Not Med Students;

AlRyalat 2019	Biomedical researchers and students knowledge about predatory journals	AlRyalat, SA; Farah, RI; Shehadeh, B; Abukeshek, A; Aldabbas, L; Al-Fawair, A; Ababneh, O	2019	Journal of Academic Librarianship	45	5		Exclusion reason: Not Med Students;
Bertoglia 2018	Predatory journals: a new threat to scientific publications	Bertoglia, MP; Aguila, A	2018	Revista Medica de Chile	146	2	206-212	Exclusion reason: Not English;
Tomlinson 2023	Analysis of predatory emails in early career academia and attempts at prevention	Tomlinson, OW	2023	Learned Publishing	36	2	156-163	Exclusion reason: Not Med Students;
Abu-Zaid 2020	Supplements to increase trainee-authored publications pertaining to medical education: A graduate's viewpoint	Abu-Zaid, A	2020	Journal of Postgraduate Medicine	66	1	35-37	Exclusion reason: Not Original Research;
Singh 2020	Analysis of Quantity and Quality of Medical Research in India: A Narrative Review	Singh, G	2020	Journal of Clinical and Diagnostic Research	14	3		Exclusion reason: Not Med Students;
Colmers-Gray 2020	Evidence-based Medicine Simulation: A Novel and Practice-relevant Approach to Teaching Real-time Literature Searching to Emergency Medicine Residents	Colmers-Gray, IN; Ha, DJ; Tan, MC; Dong, SL	2020	AEM Education and Training	4	4	428-432	Exclusion reason: Not Med Students;
Wilson 2019	Predatory journals: A warning for us all.	Wilson DJ	2019	Medical Teacher	41	12	1452	Exclusion reason: Not Original Research;
